# Possible benefits from post-mastectomy radiotherapy in node-negative breast cancer patients: a multicenter analysis in Korea (KROG 14-22)

**DOI:** 10.18632/oncotarget.16241

**Published:** 2017-03-15

**Authors:** Hae Jin Park, Kyung Hwan Shin, Jin Ho Kim, Seung Do Ahn, Su Ssan Kim, Yong Bae Kim, Won Park, Yeon-Joo Kim, Hyun Soo Shin, Jin Hee Kim, Sun Young Lee, Kyubo Kim, Kyung Ran Park, Bae Kwon Jeong

**Affiliations:** ^1^ Departments of Radiation Oncology, Hanyang University Hospital, Seoul, Korea; ^2^ Department of Radiation Oncology, Seoul National University College of Medicine, Seoul, Korea; ^3^ Department of Radiation Oncology, Seoul National University Hospital, Seoul, Korea; ^4^ Department of Radiation Oncology, University of Ulsan College of Medicine, Seoul, Korea; ^5^ Department of Radiation Oncology, Yonsei University College of Medicine, Seoul, Korea; ^6^ Department of Radiation Oncology, Sungkyunkwan University School of Medicine, Seoul, Korea; ^7^ Department of Radiation Oncology, Proton Therapy Center, National Cancer Center, Goyang, Korea; ^8^ Department of Radiation Oncology, CHA University School of Medicine, Pocheon, Korea; ^9^ Department of Radiation Oncology, Keimyung University School of Medicine, Daegu, Korea; ^10^ Department of Radiation Oncology, Chonbuk National University Hospital, Jeonju, Korea; ^11^ Department of Radiation Oncology, Ewha Womans University School of Medicine, Seoul, Korea; ^12^ Department of Radiation Oncology, Ewha Womans University Mokdong Hospital, Seoul, Korea; ^13^ Department of Radiation Oncology, Gyeongsang National University School of Medicine, Jinju, Korea

**Keywords:** breast cancer, post-mastectomy radiotherapy, risk factors

## Abstract

**Purpose:**

This study was performed to identify a subset of patients who may benefit from post-mastectomy radiotherapy (PMRT) among node-negative breast cancer patients.

**Materials and Methods:**

We retrospectively reviewed 1,828 patients with pT1-2N0 breast cancer, treated with mastectomy without PMRT from 2005 to 2010 at 10 institutions. Univariate and multivariate analyses for locoregional recurrence (LRR) and any first recurrence (AFR) were performed according to clinicopathologic factors and biologic subtypes.

**Results:**

During a median follow-up period of 5.9 years (range: 0.7-10.4 years), 98 patients developed AFR (39 isolated LRR, 13 LRR with synchronous distant metastasis, and 46 isolated distant metastasis), and 52 patients developed LRR. The 7-year LRR and AFR rates were 3.8% and 6.7%, respectively. Multivariate analysis revealed that age of ≤ 40 years (*p*<0.001) and T2 stage (*p*=0.013) were independent risk factors for LRR. The 7-year LRR rates were 2.5% with no risk factors, 4.5% with one risk factor, and 12.4% with two risk factors. Multivariate analysis for AFR revealed that age of ≤ 40 years (*p*<0.001), T2 stage (*p*<0.001), and triple-negative biological subtype (*p*=0.045) were independent risk factors for AFR. The 7-year AFR rates were 3.9% with no risk factors, 8.4% with one risk factor, and 15.7% with two to three risk factors.

**Conclusions:**

Mastectomy without PMRT is a sufficient local treatment for pT1-2N0M0 breast cancer. Nevertheless, PMRT might be considered for patients with two or three risk factors, among those of young age, with T2 tumors, and with the triple-negative biological subtype based on LRR and AFR.

## INTRODUCTION

There is a consensus that post-mastectomy radiotherapy (PMRT) is indicated for the breast cancer with locally advanced disease (T3-T4), or four or more positive axillary lymph nodes (LNs) [1, 2]. Additionally, based on the results of a recent meta-analysis [3], PMRT can be applied to one to three positive axillary LNs.

On the other hand, mastectomy is generally considered a sufficient local treatment method for node-negative breast cancer. Nevertheless, a high rate of local recurrence has been reported in a subset of patients with aggressive clinicopathologic factors such as young age, large tumor size, high tumor grade, lymphovascular invasion (LVI), or positive/close resection margin [4]. As the understanding of the molecular biology of breast cancer improves, the biological subtype is receiving attention as a possible prognostic factor with which to distinguish patients with a high *versus* low risk of local recurrence [5–7]. In the setting of mastectomy, however, the effects of the biological subtype on local recurrence are not consistent [5, 8–10].

The purpose of this study was to identify the risk factors for locoregional recurrence (LRR) and any first recurrence (AFR) in node-negative patients treated with mastectomy but not PMRT, thus defining a subgroup of patients who may benefit from PMRT.

## RESULTS

### Patient and tumor characteristics

The median follow-up period was 5.9 years (range: 0.7-10.4 years). Table 1 summarizes the patient and tumor characteristics. Adjuvant systemic treatment was delivered at the physician's discretion. A total of 182 patients (10.0%) received no systemic treatment. A total of 966 patients (52.8%) received adjuvant chemotherapy; 772 received an anthracycline-based regimen; 126 received a combination regimen comprising cyclophosphamide, methotrexate, and 5-fluorouracil; and only 31 received a taxane-containing regimen. A total of 1,260 patients (68.9%) received endocrine treatment. Among 467 HER2+ patients, only 107 (22.9%) were confirmed to have been treated with trastuzumab (not done, *n* = 292; unknown, *n* = 68). Among 255 triple negative (TN) patients, 193 (75.7%) were treated with cytotoxic chemotherapy (not done, *n* = 62).

**Table 1:** Patient, tumor, and treatment characteristics (*n* = 1,828)

Variables	*n*	%
Age, median (range)	51 (22-87)	
Menopausal status		
Premenopausal	852	46.6
Postmenopausal	848	46.4
Unknown	128	7.0
Histology		
IDCa	1695	92.7
Others	133	7.3
T stage		
T1	1141	62.4
T2	687	37.6
Tumor histologic grade		
Low-intermediate	1076	58.9
High	671	36.7
Unknown	81	4.4
Resection margin		
Negative (≥2mm)	1400	76.6
Close (<2mm)	415	22.7
Positive	11	0.6
Unknown	2	0.1
Axillary management		
SLNBx	912	49.9
ALND	914	50.0
Not done	2	0.1
Number of nodes examined		
Median, SLNBx	4 (0-43)	
Median, ALND	11 (1-48)	
<10	1157	63.3
≥10	662	36.2
Unknown	9	0.5
Hormonal receptor		
Positive	1265	69.2
Negative	563	30.8
HER2 status		
Positive	467	25.5
Negative	1189	65.0
Unknown (all IHC2+, FISH not done)	172	9.4
Systemic treatment		
Endocrine therapy alone	680	37.2
Chemotherapy alone	386	21.1
Both	580	31.7
Neither	182	10.0
Trastuzumab on HER2(+)		
Trastuzumab(+)	107	5.9
Trastuzumab(-)	292	16.0
unknown	68	3.7
Biological subtype		
Luminal A	719	39.3
Luminal B	162	8.9
Luminal HER2	201	11.0
HER2+	266	14.6
Triple negative	255	13.9
Unknown	225	12.3

Abbreviations: IDCa = infiltrating ductal carcinoma; SLNBx = sentinel lymph node biopsy; ALND = axillary lymph node dissection; IHC = immunohistochemical staining; FISH = fluorescence *in situ* hybridization.

### Locoregional recurrence

Of all 1,828 patients, 52 developed LRR. There were 31 (59.6%) ipsilateral chest wall recurrences, 28 (53.8%) ipsilateral LN recurrences (axillary, 18; internal mammary, 7; supraclavicular, 8; site unspecified, 2), and 7 (13.4%) ipsilateral chest wall and nodal recurrences. The cumulative rates for LRR at 5, 7, and 10 years were 2.8%, 3.8%, and 3.8%, respectively.

When performing univariate and multivariate analysis, patients with unknown information were not included, and the factors associated or marginally associated with each end point by univariate analysis were subject to multivariate analysis.

On univariate analysis, an age of ≤ 40 years (*p* < 0.001) and T2 stage (*p* = 0.006) were significantly associated with a high risk of LRR, and the biological subtype was marginally associated with high LRR. With respect to the biologic subtypes, the 7-year cumulative incidence of LRR was 2.2% for luminal A, 7.0% for luminal B, 5.1% for luminal HER2, 4.4% for HER2+, and 5.1% for TN (*p* = 0.095). The use of trastuzumab in patients with HER2+ did not affect LRR (Table 2). On multivariate analysis (Table 3), an age of ≤ 40 years (HR, 3.3; *p* < 0.001) and T2 stage (HR, 1.3; *p* = 0.013) were independently associated with a high risk of LRR.

**Table 2:** Univariate analysis for locoregional recurrence, distant metastasis, any first recurrence, and overall mortality

Variables	Total (*n*= 1828)	Locoregional recurrence (*n*= 52)	*p*	Distant metastasis (*n*= 61)	*p*	Any first recurrence (*n*= 98)	*p*	Any death (*n*= 44)	*p*
Age									
≤40 years	255	17	0.000	14	0.053	28	0.000	7	0.721
>40 years	1573	35		47		70		37	
T stage									
T1	1141	23	0.006	20	0.000	40	0.000	19	0.011
T2	687	29		41		58		25	
Resection margin									
Negative	1400	35	0.124	45	0.620	70	0.240	33	0.910
Close+positive	426	17		16		28		11	
Unknown *	2	0		0		0		0	
Axillary dissection									
SLNBx	912	25	0.994	25	0.273	43	0.404	22	0.481
ALND	914	27		36		55		22	
not done *	2	0		0		0		0	
Chemotherapy									
Yes	966	30	0.642	44	0.006	65	0.017	24	0.941
No	862	22		17		33		20	
HER2 status									
Negative	1189	36	0.637	44	0.763	70	0.701	25	0.205
Positive & trastuzumab(+)	107	2		2		4		1	
Positive & trastuzumab(-)	292	11		11		19		10	
Positive & trastuzumab(unknown) *	68	1		3		3		3	
Unknown *	172	2		1		2		5	
Tumor histologic grade									
Low-intermediate	1076	26	0.259	27	0.009	46	0.011	19	0.026
High	671	22		32		47		23	
Unknown *	81	4		2		5		2	
Biological subtype									
Triple negative	255	12	0.075	16	0.011	24	0.006	12	0.009
Others	1401	38		44		72		27	
Unknown *	172	2		1		2		5	
Luminal A	719	14	0.095	15	0.004	25	0.000	7	0.011
Luminal B	162	8		11		18		5	
Luminal HER2	201	7		8		12		6	
HER2+	266	7		8		14		8	
Triple negative	255	12		16		24		12	
Unknown *	225	4		3		5		6	

Abbreviations: SLNBx = sentinel lymph node biopsy; ALND = axillary lymph node dissection

* Patients with unknown information were not included in statistical analyses.

**Table 3:** Multivariate analysis for locoregional recurrence, any first recurrence, and distant metastasis

Variable	Locoregional recurrence		Distant metastasis		Any first recurrence	
	Hazard ratio (95% CI)	*p*	Hazard ratio (95% CI)	*p*	Hazard ratio (95% CI)	*p*
Age (>40 years *vs*. ≤40 years)	3.290 (1.792-6.041)	0.000	1.951 (1.068-3.563)	0.030	2.606 (1.661-4.090)	0.000
T stage (1 *vs*. 2)	1.279 (1.053-1.554)	0.013	1.482 (1.234-1.779)	0.000	1.304 (1.132-1.502)	0.000
Biologic subtype (others vs. triple negative)		-		-	1.632 (1.011-2.636)	0.045
Tumor grade (low-intermediate vs. high)		-		-		-
Chemotherapy (yes *vs*. no)		-		-		-

Abbreviations: CI = confidence interval

### Distant metastasis

Sixty-one patients developed distant metastasis (DM). Thirteen patients developed DM with synchronous LRR, and two patients developed DM 6 and 11 months after LRR. Forty-six patients had isolated DM. The cumulative rates for DM at 5, 7, and 10 years were 3.2%, 4.1%, and 5.3%, respectively.

### Any first recurrence

In total, 98 cases developed AFR; 13 LRR with synchronous DM, 39 LRR without DM, and 46 DM only. Additional two DM was reported after the first recurrence, and the total cases of DM were 61. The cumulative rates for AFR at 5, 7 and 10 years were 5.3%, 6.7% and 7.9%, respectively. On univariate analysis (Table 2), an age of ≤ 40 years (*p* < 0.001), T2 stage (*p* < 0.001), a high tumor grade (*p* = 0.011), and biological subtype (*p* < 0.001) were significantly associated with a high risk of AFR. With respect to the biologic subtype, the 7-year cumulative incidence of AFR was 4.4% for luminal A, 13.9% for luminal B, 8.2% for luminal HER2, 7.2% for HER2+, and 9.1% for TN (*p* < 0.001). The use of trastuzumab in patients with HER2 and luminal HER2 subtypes was not associated with AFR (Table 2). To facilitate comparison between the groups in the multivariate analysis, these five subtypes were redefined as binary variables based on the results of the univariate analysis: TN tumors and others (*p* = 0.006). The use of chemotherapy was significantly associated with a high risk of AFR (*p* = 0.017), and this was attributed to the development of DM as a first recurrence. On multivariate analysis (Table 3), an age of ≤ 40 years (HR, 2.6; *p* < 0.001), T2 stage (HR, 1.3; *p* < 0.001), and the TN biological subtype (HR, 1.6; *p* = 0.045) were independently associated with a high risk of AFR.

### Overall mortality

In total, 44 patients died, and 29 of them had developed tumor recurrence before death. The cumulative rates for overall mortality at 5, 7, and 10 years were 1.8%, 2.9%, and 3.6%, respectively.

### Identification of patients at high risk of LRR and AFR

To identify patients at higher risk of recurrence, we defined subgroups according to risk factors proven on multivariate analysis for LRR and ARF.

**Figure 1:**
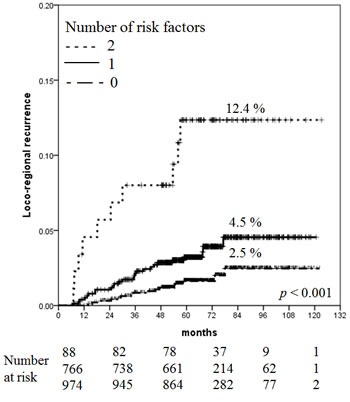
Increased risk of LRR with increasing number of risk factors

For LRR, patient age ( ≤ 40 *vs*. > 40 years) and tumor size (T1 *vs*. T2) were used to stratify the risk groups. Of all 1,828 patients, 974 (53.3%) had no risk factors, 766 (41.9%) had one risk factor, and 88 (4.8%) had two risk factors. The 5-year cumulative LRR rates were 1.6% with no risk factors, 3.2% with one risk factor, and 12.4% with two to three risk factors. The latter was defined as the high-risk group for LRR. The 7-year and 10-year cumulative LRR rates were 2.5% with no risk factors, 4.5% with one risk factor, and 12.4% with two risk factors (Figure 1).

**Figure 2:**
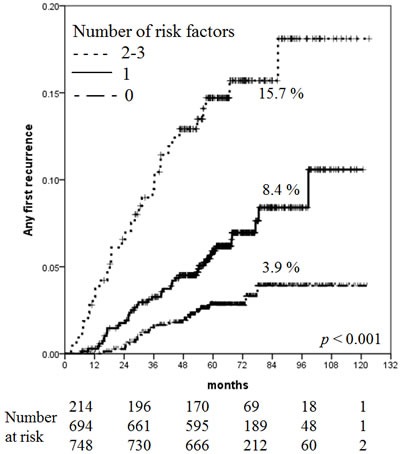
Increased risk of AFR with increasing number of risk factors

For ARF, three risk factors were used to stratify the risk groups: patient age, tumor size, and biological subtype. Of 1,656 analyzable patients (172, unknown), 748 (45.2%) had no risk factors and 694 (41.9%), 194 (11.7%), and 20 (1.2%) patents had one, two, and three risk factors, respectively. Because of the small number of patients with three risk factors, patients with two to three risk factors were assigned to the high-risk group (214, 12.9%). The 5-year cumulative ARF rates were 2.8% with no risk factors, 5.9% with one risk factor, and 14.7% with two to three risk factors. The 7-year cumulative ARF rates were 3.9% with no risk factors, 8.4% with one risk factor, and 15.7% with two to three risk factors (Figure 2). The 10-year cumulative ARF rates were 3.9% with no risk factors, 10.6% with one risk factor, and 18.1% with two to three risk factors.

### The effect of chemotherapy regimen

We performed an additional analysis comparing anthracycline-based chemotherapy (772 out of 966, 80%) and other regimens. There was no difference in LRR (*p* = 0.150), DM (*p* = 0.543), AFR (*p* = 0.829), and OS (*p* = 0.334) between two groups.

## DISCUSSION

The present study showed that the 7-year overall incidence of LRR was 3.8% in patients with pT1-2N0 breast cancer treated with mastectomy but not PMRT. Additionally, the 7-year LRR rate of patients aged less than or equal to 40 years with T2 tumors, who were considered at high risk, was 12.4%. Since no LRR was reported after 7 years, the LRR rate at 7-year and 10-year were identical (Figure 1). These results are in agreement with those of recent studies (Table 4).

**Table 4:** Recurrences in pT1-2N0 patients treated with mastectomy without post-mastectomy radiotherapy (studies performed after 1990s) [4]

					10-year locoregional recurrence rate		10-year Any first recurrence rate			
author	year	*n*	Median follow-up		Overall	High risk group		Overall	High risk group	Definition of high risk
Truong [16]	1989-1999	1505	7.0 Years		7.8%	21.2%		-	-	LVI, Grade 3
Yildirim [12]	1990-2004	502	6.4 Years	≤40 Years	7.0% (6.4 Years)	53.0%		19.0% (total)	-	LVI, size >2 cm
				>40 Years	1.7% (6.4 Years)	33.0%			-	LVI, size >3 cm, Grade 3
Sharma [11]*	1997-2002	753	7.5 years		2.1%	18.6%		8.6%	-	T2N0, age ≤40 years
Truong [10]	1998-2009	1994	4.3 Years		1.8%-3.1% (5 Years)	12.5% (5 Years)		-	-	TN, close/positive RM
Our study	2005-2011	1828	6.9 Years		3.8%	12.4%				LVI, size >2 cm
								7.9%	18.1%	LVI, size >2 cm, TN

Abbreviations: LVI = lympho-vascular invasion; TN = tripe negative; RM = resection margin

* Patients with pN1 were included in the study.

The indications for PMRT have traditionally been based on the risk of LRR. Although numerous studies have reported the prognostic value of clinicopathologic factors predicting LRR, such as patient age, menopausal status, tumor size, tumor grade, LVI, or resection margin status, variation exists in terms of the significance or magnitude of those factors in the setting of mastectomy without PMRT for pT1-2N0. Among them, young age and large tumor size, which were proven as significant prognostic factors in our analysis, are widely accepted variables associated with a high rate of LRR. With respect to patient age, Sharma et al. [11], Karlsson et al. [6], and Yildirim et al. [12] identified an age of ≤ 40 years as an independent predictor of high LRR, and Abi-Raad et al. [13] and Wallgren et al. [14] reported ages of ≤ 50 and ≤ 60 years as cutoffs, respectively. Similarly, Jagsi et al. [15] showed that a premenopausal status was an independent predictor of LRR. Regarding tumor size, T2 tumors (≥ 2 cm) were the most commonly reported cutoff point predicting a high risk of LRR [14–16].

Although the EBCTCG adopted AFR (irrespective of LRR or DM) as the primary endpoint for the effect of RT in patients with breast cancer [17], there are little data regarding AFR in mastectomy without PMRT among patients with pT1-2N0 cancer. We performed an analysis using AFR as another primary endpoint and stratified the risk groups based on three factors proven to be significant in the multivariate analysis: age of ≤ 40 years, T2 tumors, and TN subtype. The 10-year AFR rate of the high-risk groups with two to three adverse factors was 18.1%. There are scarce data with which to directly compare our findings.

Notably, the EBCTCG meta-analysis reported that LRR and AFR occurred more frequently in the no-RT group treated with axillary sampling than in the no-RT group treated with axillary dissection; however, the difference disappeared when PMRT was added, irrespective of axillary management. In contrast, we found no difference in the LRR or AFR rate according to axillary management. Moreover, the 10-year LRR and AFR rates were far superior to the outcomes of the EBCTCG in node-negative patients (the 10-year AFR and LRR rates were 7.9% and 3.8%, respectively). Even for the high-risk group defined in our study, the 10-year AFR and LRR rates were 18.1% and 12.4%, respectively. Considering the period of patient enrollment (1964-1986 in the EBCTCG meta-analysis *vs*. 2005-2011 in our study), it is reasonable that this reduction in recurrence is attributable to improved nodal examination, such as sentinel LN detection or pathologic evaluation, and progress in systemic treatments such as HER2 targeted therapy. Importantly, routine use of systemic treatment, which was given to 90% of our study cohort, could also explain these superior outcomes, in that recent large clinical trials emphasized the impact of systemic treatment in terms of not only DM and survival, but also local control [18–20].

We defined the high-risk group as described above and found a high rate of recurrence in that subset. The remaining question is how many recurrences justify the recommendation for PMRT. Olivotto and Truong suggested that PMRT is indicated when LRR exceeds 25%, but not when it is < 10%, based on the magnitude of absolute LRR reduction and the absolute survival benefit (4:1 ratio according to the EBCTCG) [21]. If the LRR rate is 12.8% (the 10-year rate in our high-risk group), patients’ priorities and preferences should be considered when making decisions regarding PMRT. The EBCTCG suggested a new ratio: for every 1.5 patients in whom AFR is avoided at 10 years, there is an additional survivor at 20 years [17]. This works well for pT1-2N1 patients with mastectomy, but not for pT1-2N0 patients, as previously described [3]. Despite the reduction in AFR by PMRT (34.2% in no-RT *vs*. 22.1% in RT at 10 years, 2*p* = 0.0003) in patients who underwent mastectomy and only axillary sampling, breast cancer mortality did not differ (35.8% in no-RT *vs*. 32.0% in RT at 20 years, 2*p* > 0.1). Even if PMRT reduced the AFR rate of 18.1% (the 10-year rate in our high-risk group) to a certain degree, it is reasonable that the absolute survival benefit would be small. This small survival benefit cannot be considered as grounds for routine use of PMRT for all high-risk patients with pT1-2N0 cancer. However, considering that most patients in our high-risk group were aged ≤ 40 years, even a small survival benefit may be important. Furthermore, the reduction in LRR or AFR would enable patients to work and live without a disease burden.

It is important to recognize the limitations of this study, including those inherent to retrospective studies. First, the follow-up period was not long enough to show all recurrences considering the long natural history of breast cancer. This might weaken the study's statistical power. Second, our study could not directly address the survival benefits in association with reductions in the LRR or AFR by PMRT, because our data regarding patient death included all-cause mortality rather than breast cancer mortality. Despite these limitations, our study is clinically valuable. Although breast-conserving treatment has been generalized for node-negative breast cancer over the past several years, some patients still undergo mastectomy for various reasons, such as the presence of multifocal breast cancer or the patient's preference for breast reconstruction after mastectomy. However, most such reference studies were performed from the 1960s to the 1990s. The present work was a multi-institutional study performed in the 2000s and included a large number of patients with pT1-2N0 breast cancer (*n* = 1,828) treated with mastectomy without PMRT. The conclusions that can be drawn from our analyses are more relevant to contemporary practice, in that we adopted current diagnostic and therapeutic strategies.

This analysis was performed only in the population who did not receive radiation after mastectomy, prospective study to compare those who received PMRT to those who did not is being planned. Furthermore, the study regarding hypofractionated RT after mastectomy could be considered. Some investigators have suggested that hypofractionated RT is also an alternative option for PMRT [22], although it has not been thoroughly studied as in post-breast conserving surgery RT [23–25].

In conclusion, our study found that the overall recurrence and LRR rates were substantially low in patients with pT1-2N0 breast cancer treated with mastectomy and systemic therapy without PMRT. This finding shows that mastectomy without PMRT is a sufficient local treatment for pT1-2N0M0 breast cancer. However, there was a patient group at high risk for recurrence: the 7-year LRR rate in patients aged ≤ 40 years with T2 tumors was 12.4%, and the 7-year AFR rate of patients with two to three adverse factors, among those aged ≤ 40 years, T2 tumors and the TN subtype, was 15.7%. PMRT might be considered for these high-risk patients.

## MATERIALS AND METHODS

This multi-institutional retrospective study was approved by the Korean Radiation Oncology Group (KROG 14-22) and the Institutional Review Board of each of 10 participating institutions in Korea. After obtaining this approval, we reviewed the medical records of patients with breast cancer treated by mastectomy from 2005 to 2010. The eligibility criteria were (1) a tumor size of ≤ 5 cm (pT1 and pT2), (2) negative LNs (pN0) proven by axillary dissection or sentinel LN biopsy, and (3) no treatment with adjuvant PMRT. The exclusion criteria were (1) male gender, (2) the presence of distant metastasis (DM) at diagnosis, (3) neoadjuvant systemic treatment, (4) a history of radiotherapy (RT) to the chest or neck, (5) a history of malignancies other than papillary/follicular thyroid cancer, and (6) bilateral breast cancer. We identified 1,842 patients according to these eligibility criteria. We then excluded 14 patients who were lost to follow-up < 6 months from the mastectomy date. Finally, 1,828 patients with breast cancer were included in this study.

The collected clinicopathological information was presented in Table 1. Positivity of ER, PR, HER2, and Ki-67 was determined by immunohistochemical staining. HER2-positivity was defined as a 3+ immunohistochemical result or a 2+ immunohistochemical result, confirmed by fluorescence *in situ* hybridization. Using the histologic grade as a surrogate for Ki-67 based on the St. Gallen Expert Consensus [14], we approximated five breast cancer subtypes based on hormone receptor (ER and PR) status, HER2 status, and histologic grade: luminal A (ER+ or PR+/HER2−/low-intermediate grade), luminal B (ER+ or PR+/HER2−/high grade), HER2+ (ER−/PR−/HER2+), luminal HER2 (ER+ or PR+/HER2+), and triple-negative (TN) (ER−/PR−/HER2−).

The primary endpoints were AFR and LRR. AFR was defined as the first tumor recurrence, irrespective of LRR or DM. LRR was defined as any LRR as a first event with or without synchronous DM. Diagnosis of DM within 3 months of an LRR was considered synchronous. LRR occurring after DM was not considered as an LRR event. LRR indicated tumor recurrence in the ipsilateral chest wall; ipsilateral axillary, infraclavicular, internal mammary, or supraclavicular node recurrence; or a combination of these. DM indicated tumor recurrence outside regions identified as LRR sites. The secondary endpoints were DM and overall mortality. The information on date of death was taken from Korea's national database, in which death by breast cancer is not distinguished from death by other causes. Time to any recurrence or death was measured from the date of mastectomy.

Cumulative incidence function curves for AFR, LRR, DM, and overall mortality were constructed using the Kaplan-Meier method, and comparisons between groups were performed using log-rank tests. A Cox proportional hazards model was used to estimate hazard ratios (HRs), and to identify correlations between outcomes and risk variables. All statistical analyses were carried out with SPSS version 18.0 (SPSS Inc., Chicago, IL, USA). *P* values < 0.05 were considered statistically significant.
